# Notes

**Published:** 1904-10

**Authors:** 


### NOTES.

It is composed of borate of sodium, albumen, carbolic acid, and glycerine, together with the crystallized principles of thyme, eucalyptus, gualtheria and mentha.

Samples can be secured by addressing J. S. Tyree, Washington, D. C.

